# Combined Impacts of Nitrogen Forms, Rice Husk Biochar, and Water Regime on Purple Rice Yield and Grain Quality

**DOI:** 10.3390/biology15040349

**Published:** 2026-02-17

**Authors:** Rachanat Limsomnuek, Supapohn Yamuangmorn, Rotsukon Jawana, Suthaphat Kamthai, Montri Sanwangsri, Chanakan Prom-u-thai

**Affiliations:** 1 Department of Plant and Soil Sciences, Faculty of Agriculture, Chiang Mai University, Chiang Mai 50200, Thailand; rachanat_lim@cmu.ac.th; 2 Lanna Rice Research Center, Chiang Mai University, Chiang Mai 50100, Thailand; supapohn.y@cmu.ac.th (S.Y.); suthaphat.k@cmu.ac.th (S.K.); 3 Energy Research and Development Institute-Nakornping, Chiang Mai University, Chiang Mai 50200, Thailand; rotsukon.j@cmu.ac.th; 4 Division of Packaging Technology, School of Agro-Industry, Faculty of Agro-Industry, Chiang Mai University, Chiang Mai 50100, Thailand; 5 Highland Agriculture and Natural Resources, Faculty of Agriculture, Chiang Mai University, Chiang Mai 50200, Thailand; montri.s@cmu.ac.th

**Keywords:** plant physiology, combined factor management, rice waste management, sustainable rice farming, nutritional values

## Abstract

Purple rice contains beneficial bioactive compounds, but the concentrations can be influenced by the growing conditions. This study investigated the interactive effects of water regime, biochar amendment, and nitrogen (N) sources on the yield and grain quality of purple rice. Purple rice grown under flooded conditions combined with biochar and urea or ammonium demonstrated significant increases in grain yield and yield components such as plant height, number of spikelets per panicle, and the percentage of filled grains compared to non-flooded conditions. Nitrate consistently resulted in the lowest yields and grain quality, especially under non-flooded conditions and with no added biochar. Grain anthocyanin concentration was highest under flooded conditions, with the maximum observed with biochar and nitrate application and with ammonium application without biochar. In contrast, the grain phenol content and antioxidant capacity were maximized by the biochar and water applications. The findings indicate that rice husk biochar can improve productivity without altering the color shade of purple rice. Combining flooding, biochar, and ammonium or urea improves the agronomic performance of purple rice, though the impact on nutritional qualities is more complex.

## Simple Summary

Purple rice is valued for its beneficial bioactive compounds, but the levels of these compounds depend on how the rice is grown. This study explored how different combinations of water management, rice husk biochar amendment, and nitrogen fertilizer types affect the yield and grain quality of purple rice. Results showed that purple rice grown under flooded conditions with added biochar and either urea or ammonium produced the highest grain yield and yield components, such as plant height, number of spikelets per panicle, and the percentage of filled grains. On the other hand, using nitrate, especially in non-flooded fields without biochar, led to the lowest yields and grain quality. Patterns of shoot nitrogen content were similar to grain yield changes. Interestingly, the highest grain nitrogen concentration was found in non-flooded conditions, regardless of whether biochar or urea/ammonium was applied. Anthocyanin content in the grain, which gives it the purple color and has health benefits, was maximized under flooded conditions, particularly when biochar and nitrate or ammonium (without biochar) were used. Grain phenol content and antioxidant capacity were highest when biochar and water were applied. Overall, using rice husk biochar can boost productivity without affecting the color shade of purple rice, but its effects on nutritional qualities are more complex.

## 1. Introduction

Purple rice (*Oryza sativa* L.) is being increasingly recognized among health-conscious consumers for its nutritional benefits, particularly its rich content of bioactive compounds such as anthocyanins, antioxidants, and essential nutrients [1,2]. The cultivation of purple rice is increasing, especially among Asian countries (e.g., China, Korea, Japan, Thailand, and Laos), where purple rice is used as a component of pharmacopeia for purposes such as improving blood circulation, kidney function, eyesight, the healing of broken bones, and skin anti-aging [3,4]. Currently, a number of new purple rice varieties have been bred in many countries, confirming that the worldwide demand for purple rice is continuously increasing [5,6]. As global interest in functional foods and sustainable agriculture grows, enhancing the productivity and grain quality of purple rice has become a key research priority.

Biochar, a carbon-rich byproduct derived from biomass pyrolysis, has gained attention as a soil amendment, including rice husk biochar, due to its potential to improve soil fertility, enhance water retention, and reduce N_2_O formation while improving nutrient use efficiency and productivity [7,8]. The application of biochar in combination with nitrogen (N) and other nutrients such as phosphorus (P) and iron (Fe) has been well documented to enhance soil health, thereby improving both grain yield and quality in rice cultivation systems [9,10,11]. Biochar significantly enhances water use efficiency. For instance, under alternate wetting and drying (AWD) irrigation, biochar application increased water productivity by up to 25.3% [12]. However, there is limited information on the effects of biochar application across different water regimes and N applications on productivity and grain bioactive compounds for the nutritional benefits in pigmented rice.

On the other hand, N plays a central role in plant growth and productivity throughout all stages of rice development, from planting to maturity [9,10]. The form in which N is applied, such as ammonium (NH_4_^+^), nitrate (NO_3_^−^), or urea, can significantly influence nutrient uptake efficiency, plant metabolism, and grain quality [11]. A recent study has shown that the appropriate combination of NH_4_^+^ and NO_3_^−^ can enhance productivity by improving soil properties, the bacterial community abundance, and N use efficiency in rice cultivation, depending on the soil type, water availability, and interactions with other soil amendments [13,14]. The effectiveness of N forms varies across different water regimes including continuous flooding, intermittent drying, and aerobic conditions, potentially influencing plant growth and grain quality. Despite growing interest in sustainable rice production using biochar for environmental benefits, there is limited research on the combined effects of N sources, husk biochar, and water regimes on rice cultivation. This gap is especially evident in purple rice, where both productivity and the enhancement of grain bioactive compounds for human health are important concerns. Therefore, it is important to investigate the integrated effects of biochar and N source applications under different water regimes on productivity and its impact on the grain bioactive compounds of purple rice. Understanding these interactions is essential for optimizing resource use efficiency and ensuring the sustainable cultivation of high-quality rice. Therefore, this study aimed to preliminarily determine the properties of rice husk biochar and to evaluate the effects of biochar amendment and N source application on the productivity and grain bioactive compounds of purple rice under varying water conditions. The findings are expected to provide insights into integrated biochar and N fertilizer management under suitable water regimes, supporting strategies to enhance the performance of nutritionally rich rice varieties.

## 2. Materials and Methods

### 2.1. Rice Husk Biochar Pyrolysis and Property Testing

Biochar was produced through slow pyrolysis at 400 ± 50 °C in a 400 L stainless steel pyrolysis reactor [15,16]. The internal temperature of the reactor increased from 24 °C to 334 °C over a period of 30 min. The purification of rice husk biochar took approximately 2 h. The cooling phase involved allowing the reactor to cool naturally until the internal temperature dropped below 50 °C. The Brunauer–Emmett–Teller (BET) surface, adsorption area, pore volume, and size were set via a high vacuum physisorption analyzer (Anton Paar, model Autosorb 6100, Anton Paar QuantaTec Inc., Boynton Beach, FL, USA). The properties of the rice husk and rice husk biochar are listed in Table 1. The scanning electron microscope (SEM) images of the rice husk and rice husk biochar were captured by using an SEM JSM-IT300 (JEOL, model JSM-IT300, JEOL Ltd., Tokyo, Japan) at 1000× magnification.

A fertilizer spreading test was conducted using 1% agar in plastic containers. The agar model was developed as an alternative to soil and/or water testing because the dark color of the rice husk biochar made it difficult to observe the movement of the fertilizer. A circular hole was made in the center of each container by carefully removing the agar. For the control treatment, a solution containing 22% urea fertilizer mixed with a dye was poured directly into the hole. For the other treatments, the urea–dye mixture was first combined with biochar, soil, or a soil + biochar mixture (75:1 *v*/*v*) before being placed into the holes. Each treatment was replicated four times. The diffusion of fertilizer was visually monitored by the movement of color through the agar at 0, 24, and 48 h after application. The diameter of the colored spread at each treatment was measured to assess fertilizer mobility.

### 2.2. Experiment Design and Rice Cultivation

The experiment was arranged in a completely randomized design with three factors (2 × 2 × 3) and four replications. The three factors consisted of two water regimes (flooded and non-flooded), two biochar amendments (with and without biochar), and three nitrogen forms (urea, ammonium, and nitrate). The pot experiment was conducted at the Lanna Rice Research Center, Chiang Mai University, Thailand (18° 47′ N, 98° 57′ E), during the rainy season (September–December) in 2024. The temperatures during the cropping seasons ranged between 20.3 and 33.4 °C, with an average temperature of 27.7 °C, 76.0% relative humidity, and average precipitation of 5.4 mm [17]. The pots were prepared with six kg of dried soil (9–10% moisture content) per pot. The pot was 30 cm in diameter and 24 cm in height. The properties of the soil used in this experiment were: a loamy sand texture, 5.07 pH, 4.28% organic matter, 4.53 ds/m electrical conductivity, 12.36 cmol (+) /kg cation exchange capacity, 0.23% total nitrogen, 85.67 mg/kg available phosphorus, and 294 mg/kg exchangeable potassium. For the added biochar treatment, rice husk biochar produced by the aforementioned process was thoroughly mixed with the soil at a rate of 80 g per pot (10 t/ha equivalent) based on a previous study of rice cultivation in Thailand [18]. The soil and biochar were mixed in a plastic bag and transferred to the pots, while for the treatment with no added biochar, an equivalent weight of soil was added directly to the pots. The purple rice variety CMU K4 was used in this experiment because it is popular among health-conscious consumers in northern Thailand. This variety is photoperiod-insensitive, with dark purple leaves, stems, and grain pericarp derived from a cross between a traditional improved variety, Kam Doi Saket (KDK), which has a purple grain color, and a modern high-yielding, non-pigmented grain variety. Rice seeds were directly sown at 10 seeds per pot. At 14 days after sowing, the seedlings were thinned to five seedlings per pot. The plants were grown under two water regimes: flooded and non-flooded conditions. For the flooded condition, the water level was kept at approximately 4.0 cm above the soil surface after the seedlings were 14 days old until maturity; for the non-flooded condition, up to 1 L of water was provided twice per day in the morning and afternoon to maintain the moisture at field capacity. Nitrogen fertilizers in three forms were applied as follows: urea (46-0-0) at a rate of 1.6 g per pot, ammonium sulfate (21-0-0) at 3.5 g per pot, and potassium nitrate (13-0-46) at 5.6 g per pot. In total, 0.72 g N/pot was applied. Fertilizer application was divided equally into three growth stages, tillering, maximum tillering, and flowering, using Zadok’s scale [19]. Phosphorus and potassium were applied as 0-52-34 and 0-0-60 fertilizers. All treatments received equal total amounts of normalized N, P, and K at 120 kg, 100 kg, and 187.7 kg per ha, respectively. Pests and weeds were managed as appropriate at each growth stage. Irrigation was withheld approximately two weeks before harvesting.

### 2.3. Sample Collection and Preparation

At maturity, plants were collected from each pot by manual harvesting for evaluation of grain yield and yield components. Seed samples from all treatments were air-dried until the moisture content reached approximately 14%. The number of tillers and panicles per plant, the number of spikelets per panicle, the number of filled grains, and the 1000-grain weight were evaluated for each replication. The percentage of filled grains was calculated from the number of filled grains and unfilled grains in each panicle. Rice straw was sampled and dried in a hot oven (100 °C for 76 h) to a constant dry weight. Seed and leaf samples were randomly collected and dried before being finely ground using a sample mill and saved for use in chemical analyses.

### 2.4. Grain Quality Analysis

Grain N concentration was analyzed using a subsample of 0.15 g of ground rice grains wrapped in aluminum foil. The total nitrogen content was analyzed using a LECO combustion furnace (LECO Co., St. Joseph, MO, USA) [20]. During the analysis, the sample was combusted at high temperature in a stream of pure oxygen, converting all N in the sample into N gas (N_2_). The amount of N gas produced was measured using the principle of thermal conductivity, by detecting changes in the gas’s heat-conducting properties. The N concentration was then calculated as a percentage of the sample weight. The total shoot N content was calculated by multiplying shoot N concentration and dry weight.

The total anthocyanin content was determined using the modified pH differential method of Abdel-Aal and Hucl [21]. About 0.5 g of freeze-dried sample was extracted with 25 mL acidified methanol (70% methanol and 30% of 1.5 mol/L HCl). Two milliliters of the supernatant was added to the two buffer solutions (0.025 mol/L potassium chloride buffer with pH 1.0 and 0.400 mol/L sodium acetate buffer with pH 4.5). The absorbance of the mixture was measured at 520 nm and 700 nm using a spectrophotometer (Biochrom Libra S22, Cambridge, UK). T-he absorbance of the diluted sample (A) was calculated as follows: A = (A520 − A700) − (A520′ − A700′), where A520 and A700 are the absorbances at 520 and 700 nm under pH 1.0, respectively, while A520′ and A700′ are the absorbances at 520 and 700 nm under pH 4.5, respectively. The absorbance of the anthocyanin pigment was expressed as cyanidin-3-glucoside, the main anthocyanin in purple rice, and was calculated as follows:

Anthocyanin = (A × MW × DF × 1000)/ε × L(1)

where A = (A520–A700 nm) at pH 1.0 − (A520′–A700′ nm) at pH 4.5; MW is the molecular weight of cyanidin-3-glucoside (449.2 g/mol); DF is the dilution factor; ε is the molar absorbance (26,900 L/mol./cm); and L is the cell pathc length (1 cm).

The total phenol concentration was determined by the modified Folin–Ciocalteu colorimetric assay of Pengkumsri et al. [22]. About 0.5 g of freeze-dried sample was extracted with 50% methanol. Then, 1 mL of the supernatant was mixed with 0.5 mL of Folin–Ciocalteu reagent and neutralized with 1.5 mL of 20% sodium carbonate (Na_2_CO_3_). The mixture was reacted at room temperature for 25 min, and the absorbance was measured at 760 nm in a spectrophotometer. A gallic acid solution (0–300 µg/mL) was used as a standard.

The antioxidant capacity was determined by the free radical scavenging activity of 2,2-diphenyl-1-picrylhydrazyl (DPPH). The DPPH assays were performed following the methods of Amarowicz et al. [23] and Benzie and Strain [24], with several modifications. Rice flour (0.1 g) was extracted with 10 mL of absolute methanol. The extract was placed on an orbital shaker (IKA KS 250 B, Profcontrol GmbH, Potsdam, Germany) for 30 min and then separated by centrifugation (MSE Super Minor, Crawley, UK) at 3396× *g* for 10 min and filtered through a 0.22 μm Nylon syringe filter. The reaction mixture contained 0.3 mL of the sample extract, 1.6 mL of methanol, and 0.5 mL of a 0.1 mmol/L DPPH solution. Blank tubes were prepared using 0.3 mL of the supernatant and 2.1 mL of methanol. The mixtures were shaken and incubated in the dark at room temperature for 20 min, and the absorbance at 517 nm was measured with a spectrophotometer. For the FRAP analysis, briefly, 2.0 mL of extracted solution was transferred to a 25 mL volumetric flask, and 20 mL of 0.1 M sodium acetate (pH 4.0), 0.5 mL of 0.5% (*w*/*v*) phenanthroline, and 0.5 mL of 0.3 mM Fe (III) were added. Blanks of the extracts were performed using samples as above, except that 0.5% (*w*/*v*) phenanthroline was not added and the samples were diluted with 0.1 M sodium acetate (pH 4.0). After incubating in a 37 °C water bath for 20 min, the absorbance at 510 nm was measured using a spectrophotometer.

### 2.5. Statistical Analysis

Data were tested for normality, and data that were not normally distributed were transformed before being subjected to analysis of variance by three-way ANOVA interaction effects using Statistix analytical software (SX for window, version 10.0, Tallahassee, FL, USA) for all measured traits. The Fisher–Snedecor F-test was used to assess the significance of sources of variability at *p* < 0.05. Duncan’s Multiple Range Test (DMRT) at a significance level of *p* < 0.05 was used to compare the mean values. Pearson correlation analysis was used to examine the relationships between parameters.

## 3. Results

### 3.1. Rice Husk and Biochar Properties

**Figure 1:**
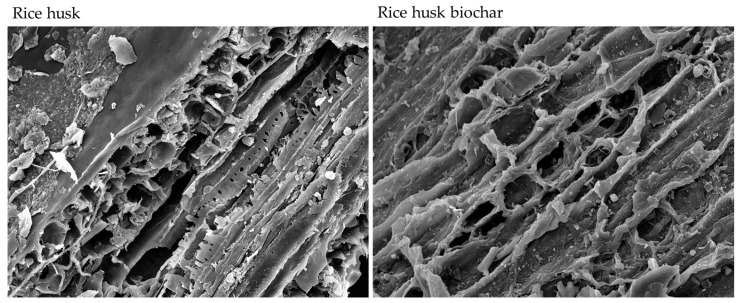
Scanning electron microscope (SEM) images of rice husk and rice husk biochar produced by slow pyrolysis at 400 ± 50 °C for 4 h at 1000× magnification.

The properties of the rice husk and rice husk biochar used in this study are shown in Table 1, and the images from the scanning electron microscope of the rice husk and rice husk biochar are shown in Figure 1. Rice husk biochar had higher concentrations of nutrients (N, P, and K) and a higher pH compared to rice husk. The predominant differences in rice husk biochar compared to rice husk were an alkaline pH (9.38), a high BET surface area (82.06 m^2^/g) and adsorption area (14.32 m^2^/g), and a measurable pore volume (0.02 cm^3^/g) and size (2.27 nm).

**Figure 2:**
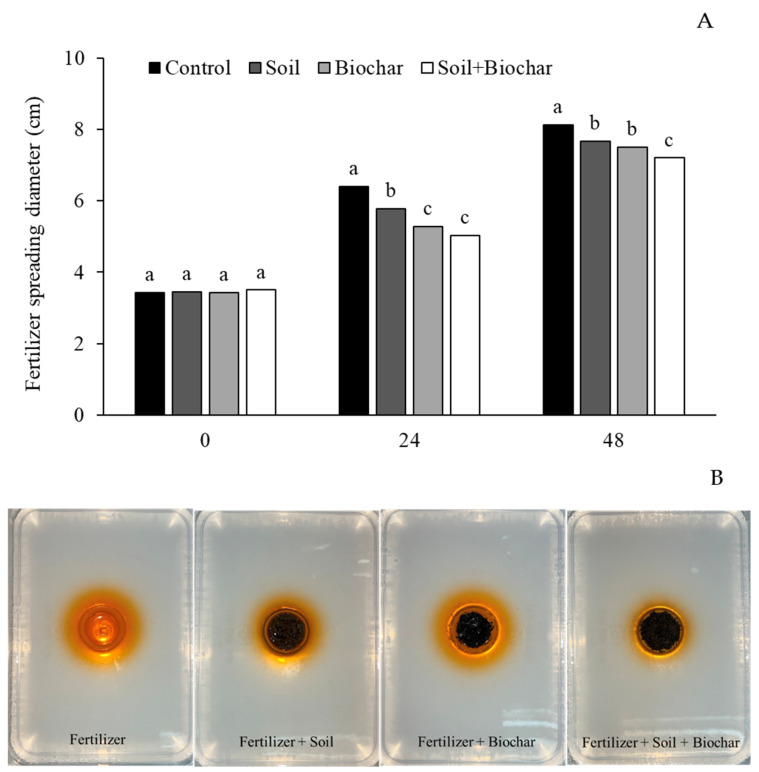
The fertilizer spreading test in 1% agar over 48 h (0, 24, and 48 h) comparing the control (fertilizer only), soil, biochar, and soil + biochar treatments (**A**), and photographs showing fertilizer-spreading characteristics in 1% agar at 48 h for fertilizer only, fertilizer + soil, fertilizer + biochar, and fertilizer + soil + biochar (**B**). Different lowercase letters above the bars indicate a significant difference by DMRT at *p* < 0.05.

The dominant properties of rice husk biochar were considered to be suitable for use as a slow-release fertilizer in rice cultivation. Therefore, the spread of the fertilizer was tested by measuring the diameter of the dyed fertilizer in 1% agar over 48 h, with comparisons made between the fertilizer alone as a control and the conditions with soil, biochar, and soil + biochar. The technique was developed to address the difficulty of observing the distribution of fertilizer when mixed with biochar, which is dark in color, during soil and water testing. The result showed that the diameter of fertilizer was initially similar among all treatments (0 h), confirming equal starting conditions, but significant differences were observed from 24 to 48 h (*p* < 0.05). The control treatment with fertilizer alone exhibited the largest diameter, while combining fertilizer with soil and/or biochar reduced the rate of spreading. Notably, the fertilizer + soil + biochar treatment consistently resulted in the smallest spreading diameter at 48 h (Figure 2A,B).

### 3.2. Grain Yield and Yield Components

**Figure 3:**
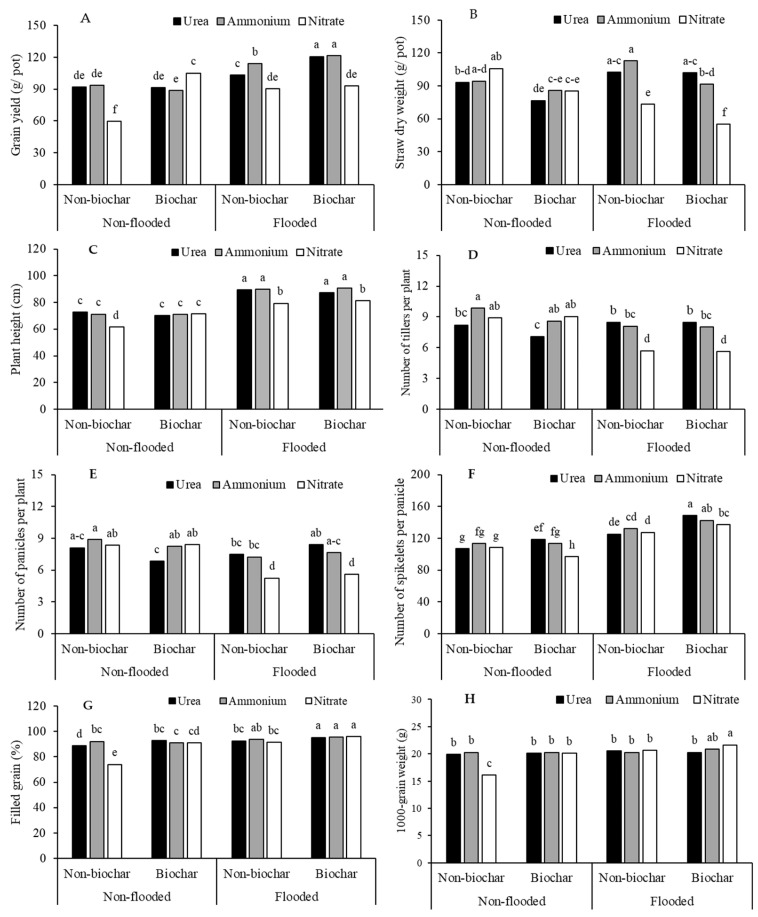
Grain yield (**A**), straw dry weight (**B**), and yield components (**C**–**H**) of purple rice grown under different applications of N forms and biochar at varying water regimes. Different lowercase letters above the bars indicate a significant difference by DMRT at *p* < 0.05.

Grain yield was significantly affected by interactions among the water regime, biochar amendment, and N form, while straw dry weight was affected by an interaction between water regime and N form (*p* < 0.05) (Table 2) (Figure 3A,B). Flooded conditions consistently produced higher grain yields and straw dry weight than non-flooded conditions. Among N forms, urea and ammonium led to significantly greater yields and straw dry weight than nitrate under most conditions (*p* < 0.05). Biochar amendment further enhanced yield, particularly when combined with flooding and either urea or ammonium, resulting in the highest grain yields (*p* < 0.05). In contrast, nitrate application, especially under non-flooded conditions without biochar, resulted in the lowest values for both grain yield and straw dry weight.

The yield component analysis revealed significant effects of water regime, biochar amendment, and N form; filled grain percentage and 1000-grain weight were especially affected by an interaction between the three factors (*p* < 0.05) (Table 2) (Figure 3C–H). Flooded conditions substantially increased plant height, the number of spikelets per panicle, the filled grain percentage, and 1000-grain weight compared to non-flooded conditions. Biochar amendment enhanced plant height and 1000-grain weight, particularly under flooded conditions. Urea and ammonium generally resulted in higher plant height and greater numbers of tillers and spikelets per panicle compared to nitrate. The highest values for most yield components were observed under flooded, biochar-amended conditions with urea or ammonium as the N source, while nitrate treatments consistently led to lower values across most parameters.

### 3.3. Grain N Concentration and Total Shoot Content

**Figure 4:**
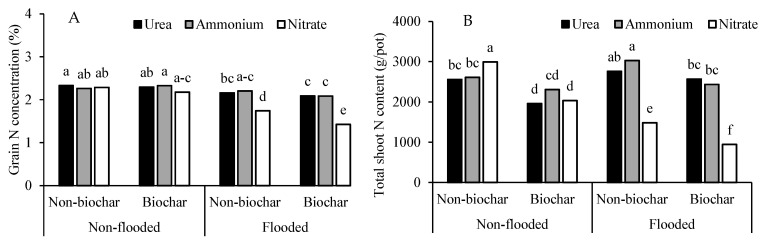
Grain N concentration (**A**) and total shoot N content (**B**) of purple rice grown under different applications of N forms and biochar with varying water regimes. Different lowercase letters above the bars indicate a significant difference by DMRT at *p* < 0.05.

Grain N concentration and total shoot N content were affected by water regime, biochar amendment, and N source; however, no interaction effect among the three factors was observed (*p* < 0.05) (Table 2) (Figure 4A,B). Grain N concentration was generally promoted by flooded conditions, particularly with urea and ammonium fertilization, while nitrate consistently resulted in lower grain N. The effect of biochar amendment was less pronounced for grain N concentration (Figure 4A). On the other hand, applying biochar substantially reduced the total shoot N content for all water regimes and N application sources, especially in nitrate form with different magnitudes of decreases. Applying biochar with all nitrogen sources under non-flooded conditions resulted in a smaller reduction in total shoot N content compared to the flooded condition (Figure 4B).

### 3.4. Grain Appearance and Quality

**Figure 5:**
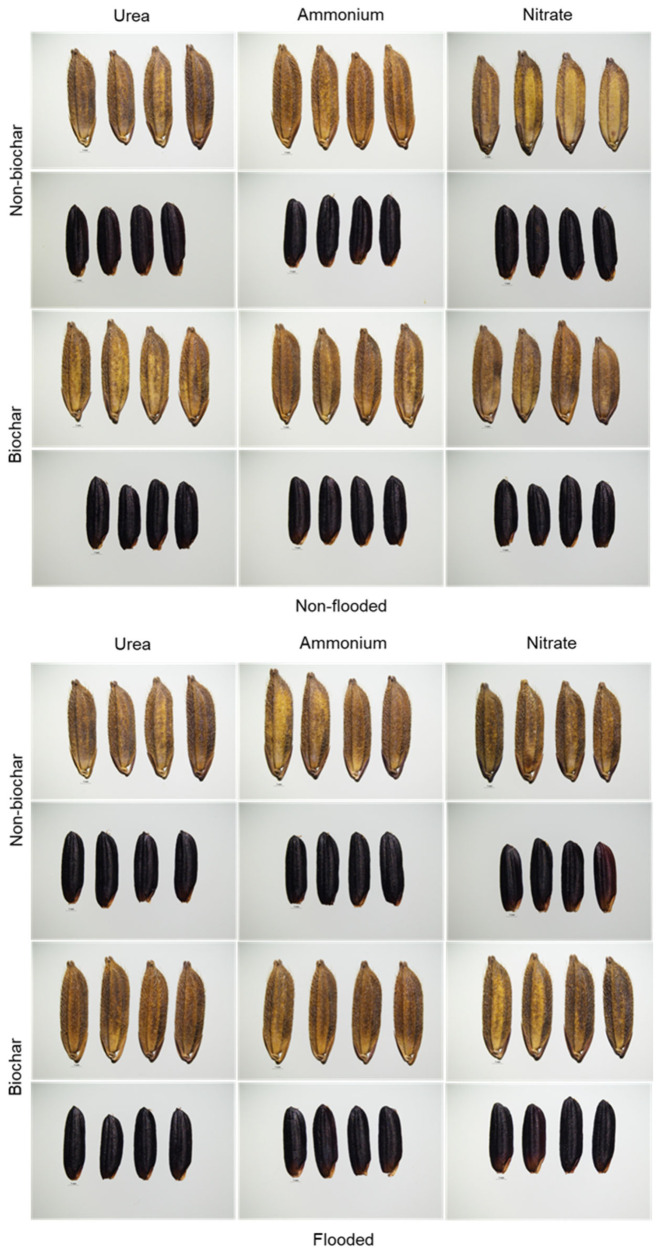
The appearance of unhusked rice and caryopsis of purple rice grown under different applications of nitrogen forms and biochar with varying water regimes.

The grain color shade, measured by a colorimeter, and grain shape, including length, width, and thickness, did not differ among the treatments in either the unhusked rice or the caryopsis (husk removed). The appearance of the rice is shown in Figure 5.

**Figure 6:**
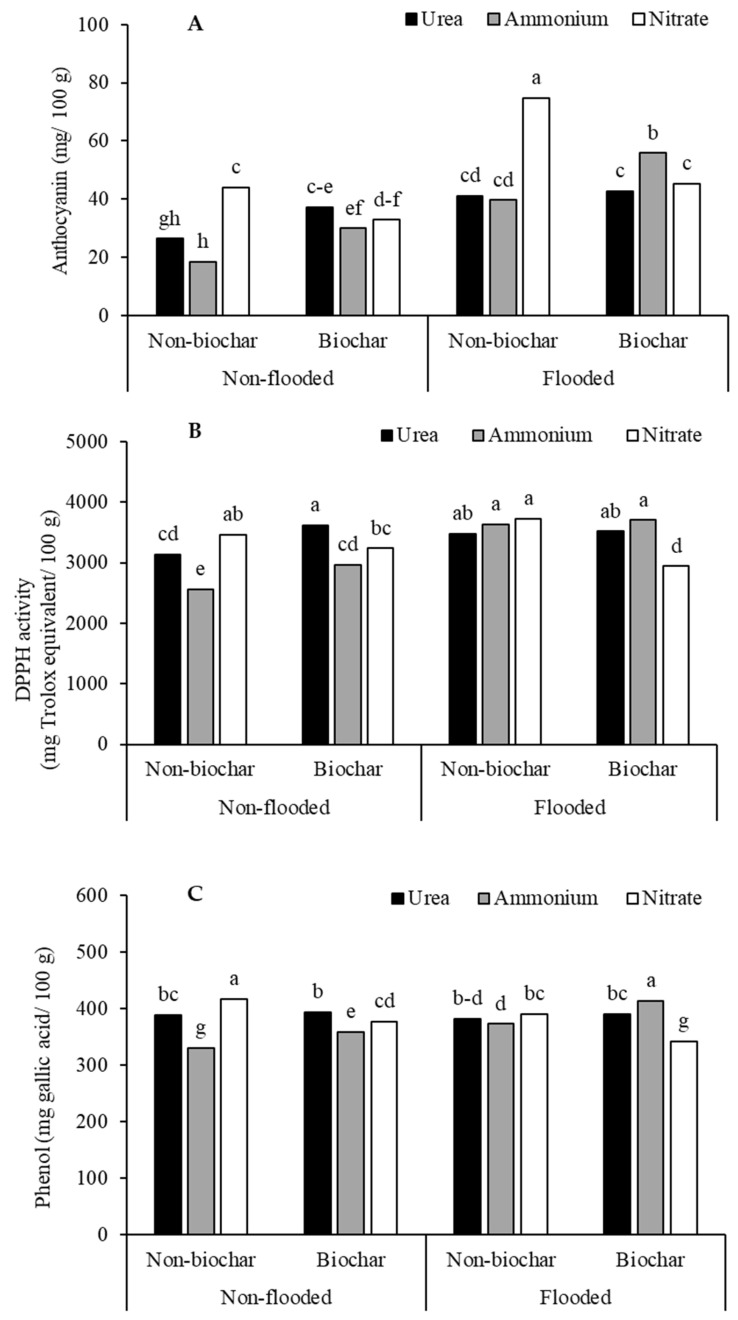
Grain anthocyanin (**A**), antioxidant activity (**B**) and phenol (**C**) concentration of purple rice grown with different applications of N forms and biochar under varying water regimes. Different lowercase letters above the bars indicate significant differences by DMRT at *p* < 0.05.

Grain bioactive compounds, including anthocyanin, DPPH radical scavenging activity, and total phenols, were significantly influenced by the water regime, biochar amendment, and N source. An interaction effect among the three factors was found for grain anthocyanin concentration (*p* < 0.05) (Table 2) (Figure 6A–C). The highest anthocyanin accumulation was observed under flooded conditions with or without biochar application. The biochar application slightly increased grain anthocyanin compared to that without biochar application under the non-flooded condition, with N sources producing a lesser effect in all conditions (Figure 6A). A similar effect was found for grain antioxidant activity (DPPH) (Figure 6B) and total phenol concentration (Figure 6C).

**Figure 7:**
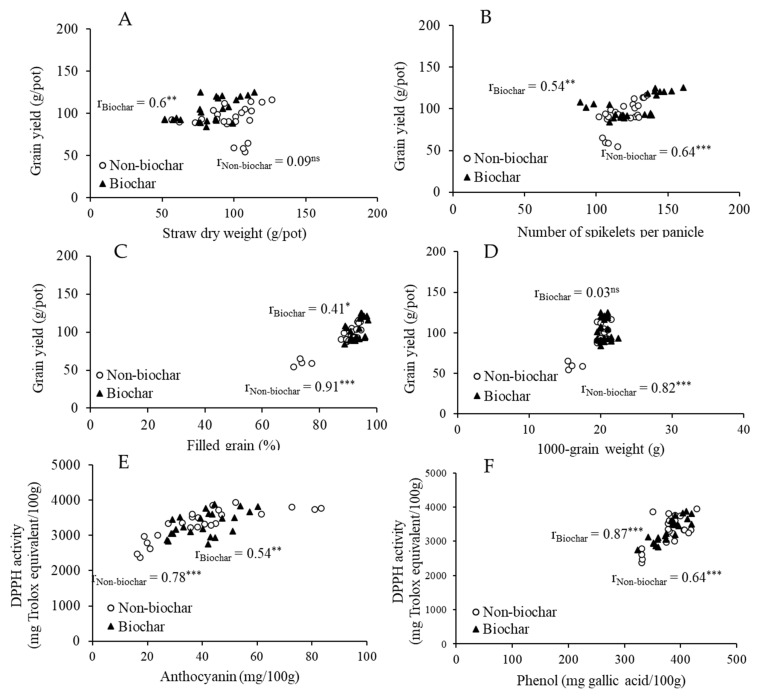
The relationship between grain yield and straw dry weight (**A**), number of spikelets per panicle (**B**), filled grain percentage (**C**) and 1000-grain weight (**D**) and the relationship between antioxidant and anthocyanin (**E**) and phenol (**F**) in the grains of purple rice grown with different applications of N forms and biochar under varying water regimes. ns indicates non-significant correlation at *p* < 0.05 and *, ** and *** indicate significant correlations at *p* < 0.05, 0.01 and 0.001, respectively.

There was a significant correlation between grain yield and straw dry weight in the biochar treatment (r = 0.60, *p* < 0.01) but not in the non-biochar treatment (Figure 7A), grain yield and number of spikelets per panicle in both the biochar (r = 0.54, *p* < 0.01) and non-biochar treatments (r = 0.64, *p* < 0.01) (Figure 7B), grain yield and percentage of filled grain in both the biochar (r = 0.41, *p* < 0.05) and non-biochar treatments (r = 0.91, *p* < 0.001) (Figure 7C), and grain yield and 1000-grain weight in the non-biochar treatment (r = 0.82, *p* < 0.001) but not in the biochar treatment (Figure 7D). There were no significant correlations between grain yield and grain N concentration or the concentrations of grain bioactive compounds across the treatments, but there were significant positive relationships between DPPH radical scavenging activity and both anthocyanin and total phenol content in rice grains (*p* < 0.05) (Figure 7E,F). Grain DPPH activity showed a strong correlation with anthocyanin content in the non-biochar treatments (r = 0.78, *p* < 0.001), while biochar treatment exhibited a moderate relationship (r = 0.54, *p* < 0.01) (Figure 7E). There was a strong correlation between grain DPPH activity and total phenolic content in the non-biochar treatments (r = 0.64, *p* < 0.001) and the biochar treatments (r = 0.87, *p* < 0.001) (Figure 7F). However, the correlation analysis results did not imply causation.

## 4. Discussion

This study has demonstrated the impacts of water regime, biochar amendment, and N source on the yield and grain quality of purple rice. Our results have shown that integrating these management practices can synergistically enhance both the agronomic performance and functional quality of rice purple rice grains.

The addition of rice husk biochar demonstrated potential benefits regarding soil nutrients, BET surface, adsorption area, pore volume, and pore size, especially for rice cultivation in areas where water is limited. Previous studies have reported that applying biochar not only regulates the availability of soil nutrients such as N, P, and K for rice cultivation through physiological and biochemical pathways but also helps to retain soil moisture by increasing soil porosity and water-holding capacity [25,26]. The study showed that soil porosity increased by 57.4% and 40.0% in soils amended with biochar + compost and with compost alone, respectively, compared to the control soil. Additionally, soil moisture content was significantly higher in the compost + biochar treatment (29%), followed by the compost alone (18.4%), while the control soil exhibited a moisture content of only 8.18%. The results of the fertilizer spreading test in the current study further confirmed that the combination of soil and biochar slowed fertilizer diffusion, as evidenced by the smallest spreading diameter over time. Previous studies indicated that synthesized fertilizer + biochar showed better performance in controlling the release of nutrients against commercial fertilizer [27,28]. After a 30-day incubation period, the soil treated with the control fertilizer released 100% of its N and P contents, whereas the lowest release was observed in the synthesized fertilizer + biochar treatment, with 69.76% for N and 70.36% for P. These results suggest that biochar’s high porosity and adsorption capacity enhance nutrient retention, reduce leaching losses, and maintain nutrient availability throughout critical growth stages in rice crop cultivation.

Additionally, biochar improved the chemical properties of soil related to pH neutralization of acidic soil by increasing the cation exchange capacity and base saturation and providing a larger surface area for the exchange of cations, thereby increasing the bioavailability of certain elements (e.g., manganese, iron, zinc, and copper) and soluble carbon in the soil, depending on the initial soil chemical properties [29,30]. Previous studies have also reported that biochar application alters soil texture fractions and increases mineral N levels, depending on soil type and climatic conditions. For example, biochar generally enhances soil inorganic N content more effectively in areas with annual rainfall between 600 and 1000 mm and temperatures above 8 °C, with the most pronounced increases observed in sandy soils and loams [31,32]. It is recommended that future studies include analysis of soil texture fractions and mineral nitrogen to strengthen the interpretation of the effects of biochar and nitrogen application in rice cultivation. The soil pH in the current study was slightly acidic (pH 5.07). The added biochar could affect soil fertility by the above chemical reaction, and applying different N sources can affect the availability of nutrients for plants. The form of N fertilizer application significantly influences N availability in soil [33,34]. Nitrate as a source of N is highly mobile and prone to leaching, which can reduce N availability for crops, whereas ammonium can be temporarily retained in the soil, enhancing N availability through adsorption on soil colloids and organic matter, and adding paddy husk compost with ammonium fertilizer has been reported to reduce leaching and increase soil N availability [33]. The current experiment showed that applying N in ammonium and urea forms, together with biochar amendment, increased soil N availability and enhanced plant growth and productivity, especially under flooded conditions. In contrast, the application of nitrate had the opposite effect, which could be explained by changes in soil pH and cation exchange capacity, as described previously. In addition, soil N availability can be influenced by microbial enzyme activity, which is affected by different N forms, thereby altering N transformation and availability. For example, urea enhances urease and protease enzyme activities, promoting N mineralization and availability [34]. The effectiveness of different N forms can vary with soil type and thus should be considered when managing biochar and N sources in purple rice cultivation. The present results suggest that while flooded conditions and the suitable N source (urea or ammonium) support higher grain N accumulation, biochar—though less impactful on grain N—can lower the overall N retained in shoots, particularly under flooding conditions. For optimal N efficiency and grain quality, it is important to carefully manage water, consider N form selection, and use biochar strategically based on specific crop goals and field conditions.

Flooded conditions consistently outperformed non-flooded regimes in grain yield and straw biomass, particularly when combined with urea or ammonium fertilization and biochar application. These results align with studies indicating the yield benefits of continuous flooding over aerobic systems for rice crop cultivation [35]. The primary mechanisms through which flooding affects zinc availability include changes in soil redox potential, pH, and interactions with other soil components. However, prolonged flooding often leads to a decrease in zinc availability as a result of the formation of stable zinc compounds. In the case of N, severe floods can cause significant losses of nitrate, especially in areas with high precipitation [36]. Biochar’s positive effects, primarily under flooded conditions, may be attributed to its ability to improve the soil structure, increase the cation exchange capacity and microbial activity, and promote the activity of iron-reducing bacteria [37]. The study found that the combined action of microorganisms and Fe (III) reduction under flooded conditions, together with biochar amendment, also promoted the availability of soil phosphorus. This suggests that biochar application should be considered, especially in crop cultivation systems exposed to continuous rainfall or field submergence, such as paddy fields. Urea and ammonium enhanced yields more effectively than nitrate, possibly due to the rice plant’s preference for ammonium-based nutrition [37,38]. In physiological and biochemical benefits, ammonium fertilization often results in higher CO_2_ assimilation rates per leaf area compared to nitrate, leading to higher Rubisco content and activity, and improved RuBP regeneration rates, which can enhance photosynthesis and overall plant growth [39]. The negative effects of nitrate were evident in both the yield and yield components, consistent with previous research suggesting rice’s inefficient nitrate utilization [40]. Across yield components, including plant height, tiller and panicle numbers, spikelet count, and grain filling, the optimal results were again associated with the combination of a flooded water regime, biochar, and ammonium or urea. Biochar further improved traits such as plant height and spikelet number, supporting the hypothesis that it enhances soil fertility and, in turn, plant development [39]. Therefore, these results indicate that optimizing water management (flooding), selecting suitable N forms (urea or ammonium), and applying biochar together can substantially improve yield and biomass production in the purple rice variety used in this study, while using nitrate as a N source in non-flooded conditions with no biochar amendment should be avoided.

Grain anthocyanin, phenol contents, and antioxidant activity (DPPH) were also influenced by biochar and water regimes, while N sources had less effect. Flooded regimes dominantly enhanced grain anthocyanin accumulation and antioxidant capacity (DPPH), consistent with previous findings that water availability influences both yield and grain quality [40]. The synthesis of anthocyanins in plants, including rice, is significantly influenced by N levels, in that low N levels tend to promote anthocyanin accumulation, while high N levels can inhibit it because N levels regulate the transcription factors involved in the anthocyanin synthesis pathway; for instance, in purple potatoes, low N levels significantly increased anthocyanin content [41]. On the other hand, it has been reported that the interaction between water and N management practices can significantly impact anthocyanin content in rice. For example, combining rainfed conditions with a high N application rate (150 kg ha^−1^) resulted in the highest anthocyanin content in colored rice [42,43]. Bioactive compounds are secondary metabolites produced by plants in response to biotic and abiotic stresses. Their synthesis is regulated by N through processes such as N uptake, allocation, and assimilation, involving key enzymes such as nitrate reductase (NR), glutamine synthetase (GS), and glutamate dehydrogenase (GDH), which are crucial for maintaining antioxidant capacity [44]. Thus, optimizing N metabolism by N fertilizer management can enhance the synthesis of these compounds during stressful conditions. This capacity is also regulated by carbon allocation within the plant, which is influenced by the supply of photosynthates and the demands of competing sink organs. Carbon allocation plays a crucial role in plant adaptation to stress. Under stressful conditions, plants adjust their carbon allocation strategies to optimize survival. For example, during drought, plants may increase carbon allocation to roots and microbial biomass to enhance water uptake and improve stress tolerance [45]. The present results showed no significant correlations between grain yield and grain N concentration or the concentrations of grain bioactive compounds across the treatments. However, the results of the correlation analysis do not imply causation. The observed relationships indicate association only, and further experimental studies are needed to determine causal links between these variables. The findings in this study indicate that while water management and biochar can enhance bioactive compounds and certain yield traits, improving grain yield does not necessarily improve grain N or bioactive compound levels. For enhanced grain quality, particularly antioxidant properties, management practices should focus on biochar application and flooding, with less emphasis on the N source, whereas maximizing yield and bioactive content may require balancing different field strategies depending on production goals. On the other hand, the results of this study may be influenced by confounding effects from accompanying ions such as sulfate (S) and potassium (K), which were applied together with different nitrogen sources, biochar, and water conditions, potentially affecting both yield performance and grain bioactive compounds, and should therefore be carefully considered. For instance, previous studies have shown that N plus S application increased rice yield by 14–16% compared to N alone by improving the leaf area index, photosynthesis rate, and enzyme activities [46], as well as enhancing health-beneficial phytochemicals like total phenolic acids in wheat [47].

For future research, it is important to further investigate the environmental implications of biochar application, particularly its role in reducing N leaching under different water regimes. Biochar has the potential to improve nutrient retention and minimize N losses to the environment, thereby decreasing the risk of groundwater contamination and greenhouse gas emissions. Examining how biochar interacts with varying water conditions (e.g., flooded versus non-flooded systems) and N fertilization will help clarify its effectiveness in promoting sustainable rice cultivation. Understanding these long-term interactions will be valuable for developing management strategies that balance high productivity with environmental protection.

## 5. Conclusions

The rice husk biochar offers potential benefits in terms of soil nutrients, BET surface area, adsorption area, pore volume, and pore size for rice cultivation. Our findings demonstrate that the amendment of biochar, especially when combined with flooded conditions and the application of ammonium or urea, enhances the yield of purple rice by approximately twofold compared to that without biochar under non-flooded conditions. This yield increase is attributed to a greater number of spikelets per panicle and a higher percentage of filled grains, without negatively affecting grain color, shade, or other appearance traits. Additionally, grain bioactive compounds in purple rice are predominantly affected by flooded conditions and biochar amendment, with less influence from N sources. These practices offer a promising approach for sustainably improving both productivity and functional values in purple rice cultivation. However, the results of this study were obtained from pot experiments conducted under controlled conditions, with limited pot and soil volume. Nevertheless, all plants appeared healthy and showed no visible signs of limiting factors across treatments. To confirm these findings and avoiding extrapolation to field conditions, further validation should be conducted under field management, involving a broader diversity of purple rice varieties.

## Figures and Tables

**Table 1:** Comparison of rice husk and rice husk biochar properties after slow pyrolysis at 400 ± 50 °C for 4 h.

Property	Rice Husk	Rice Husk Biochar	Unit
Nitrogen	0.23	0.84	%
Phosphorus	0.12	0.37	%
Potassium	0.42	1.04	%
Organic matter	62.73	10.77	%
Organic carbon	36.38	6.25	%
Carbon/nitrogen ratio	158:1	7:1	-
pH	6.16	9.48	-
Electrical conductivity	0.62	0.77	ds/m
BET surface	nd	82.06	m^2^/g
Adsorption area	nd	14.32	m^2^/g
Pore volume	nd	0.02	cm^2^/g
Pore size	nd	2.27	nm

Note: nd, not determined due to the insoluble properties of rice husk material. Brunauer–Emmett–Teller (BET) was used to determine the specific surface area of a material, which includes all external surfaces and the inner surfaces of pores.

**Table 2:** Significant effect of water regime, biochar application and nitrogen form on the yield, yield component, grain N concentration, total shoot N content, grain anthocyanin, DPPH activity and total phenol of purple rice by analysis of variance determined by Fisher–Snedecor’s F-test.

	Water Regime (W)	Biochar Application (B)	Nitrogen Form (N)	W × B	W × N	B × N	W × B × N
Grain yield	***	***	***	*	***	***	***
Straw dry weight	ns	***	***	ns	***	ns	ns
Plant height	***	ns	***	ns	*	**	ns
No. of tillers/plant	***	*	***	ns	***	ns	ns
No. of panicles/plant	***	ns	***	**	***	ns	ns
No. of spikelets/panicle	***	***	***	***	ns	***	ns
Filled grain (%)	***	***	***	***	***	***	***
1000-grain weight (g)	***	***	**	**	***	***	***
Grain N concentration (%)	***	***	***	**	***	*	ns
Total shoot N content (g/pot)	*	***	***	ns	***	ns	ns
Grain anthocyanin (mg/100 g)	***	ns	***	**	***	***	**
Grain DPPH activity (mg/100 g)	ns	ns	***	ns	***	***	ns
Grain total phenol (mg/100 g)	***	ns	**	***	***	***	ns

ns, *, **, and *** indicate non-significant and significant effects of water regime, biochar application, and nitrogen form and their interaction effects on the yield, yield component, grain N concentration, total shoot N content, grain anthocyanin, DPPH activity and total phenol of purple rice at *p* > 0.05, *p* < 0.05, *p* < 0.01, and *p* < 0.001, respectively.

## Data Availability

The data presented in this study are available on request from the corresponding author.
